# Tuberculous abscess of the left gluteal region and iliac fossa following transvaginal ultrasound-guided oocyte retrieval: a case report

**DOI:** 10.3389/fmed.2026.1785128

**Published:** 2026-03-27

**Authors:** Jiangping Wang, Qifeng Song, Zhenhao Liu, Jianan Su, Zhouyu Gao, Shenghu Zhou, Yongjie Qiao

**Affiliations:** 1 Department of Joint Surgery, The 940th Hospital of Logistics Support Force of Chinese People’s Liberation Army, Lanzhou, China; 2 Clinical Medical School of Northwest Minzu University, Lanzhou, China

**Keywords:** anti-tuberculosis treatment, assisted reproductive technology, case report, *Mycobacterium tuberculosis*, osteoarticular and soft tissue tuberculosis

## Abstract

Osteoarticular and soft-tissue tuberculosis is relatively uncommon in clinical practice and is often characterized by insidious onset and non-specific symptoms. Its manifestations may mimic those of degenerative disorders, frequently leading to delayed or incorrect diagnosis. These challenges are further amplified when the infection occurs at rare, procedure-related sites, which are easily overlooked. We report the case of a 31-year-old woman who developed persistent pain in the left gluteal and lower abdominal regions after ultrasound-guided transvaginal ovarian needle drilling (UTND) performed in May 2023. The patient subsequently experienced pregnancy loss but did not present with systemic symptoms such as fever or chills. She was initially misdiagnosed with lumbar disc herniation at an outside hospital, and conservative management failed to relieve her symptoms. Upon admission to our institution, she underwent surgical debridement of lesions in the left gluteal area and iliac fossa. Histopathological examination of intraoperative abscess specimens confirmed *Mycobacterium tuberculosis* infection, and standardized anti-tuberculosis therapy was administered postoperatively, and no recurrence has been observed during follow-up. This case highlights the need for heightened clinical vigilance regarding tuberculosis infections at rare, procedure-related anatomical sites. For patients presenting with similar non-specific symptoms, tuberculosis should be considered in the differential diagnosis to ensure timely identification and appropriate treatment, thereby preventing disease progression and serious complications.

## Introduction

Tuberculosis (TB), caused by *Mycobacterium tuberculosis*, remains a major global infectious disease despite a steady decline in incidence worldwide. It continues to pose a substantial public health burden, particularly in developing countries and among individuals with compromised immune function (1, 2). Pulmonary TB is the most common clinical presentation, whereas osteoarticular and soft-tissue tuberculosis accounts for only 1%–5% of all TB cases, making it a relatively rare entity (3–5). These forms of TB often present insidiously with non-specific symptoms and may mimic degenerative joint diseases or routine bacterial infections, resulting in significant diagnostic challenges and frequent delays or misdiagnosis (1, 6, 7). Current literature on osteoarticular and soft-tissue TB largely focuses on primary infections or cases secondary to hematogenous spread from pulmonary TB. In contrast, procedure-related localized TB infections are exceedingly uncommon, and to date, no cases of tuberculous abscess formation following ultrasound-guided transvaginal ovarian needle drilling (UTND) have been reported. With the increasing utilization of assisted reproductive technology (ART), oocyte retrieval has become a routine procedure. However, its complications primarily involve bleeding, infection, and visceral injury (8, 9), while the risk of rare complications such as post-procedural TB infection is often under-recognized. In this report, we describe a case of a left gluteal and iliac fossa tuberculous abscess that developed after UTND. By summarizing the diagnostic and therapeutic course of this patient, we aim to fill the existing gap in the literature regarding this rare procedure-related complication. Our findings serve to raise clinical awareness, provide practical diagnostic and management references, and support timely recognition and appropriate treatment to prevent severe adverse outcomes.

## Case report

**Figure 1:**
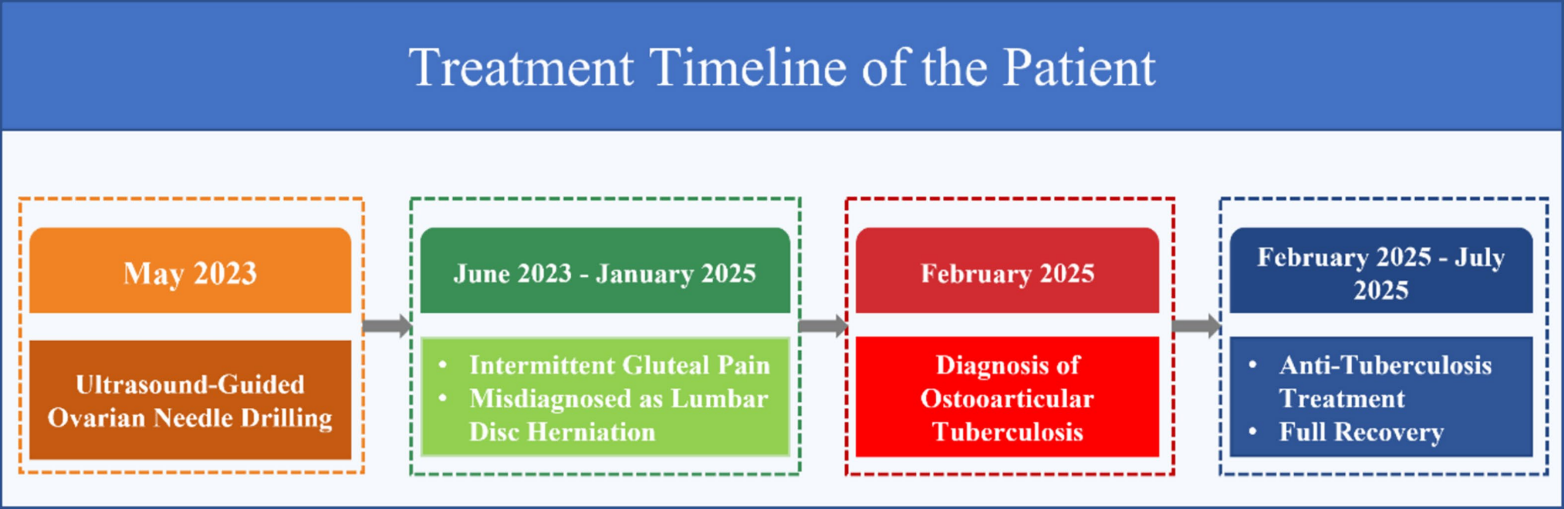
Treatment timeline of the patient.

**Figure 2:**
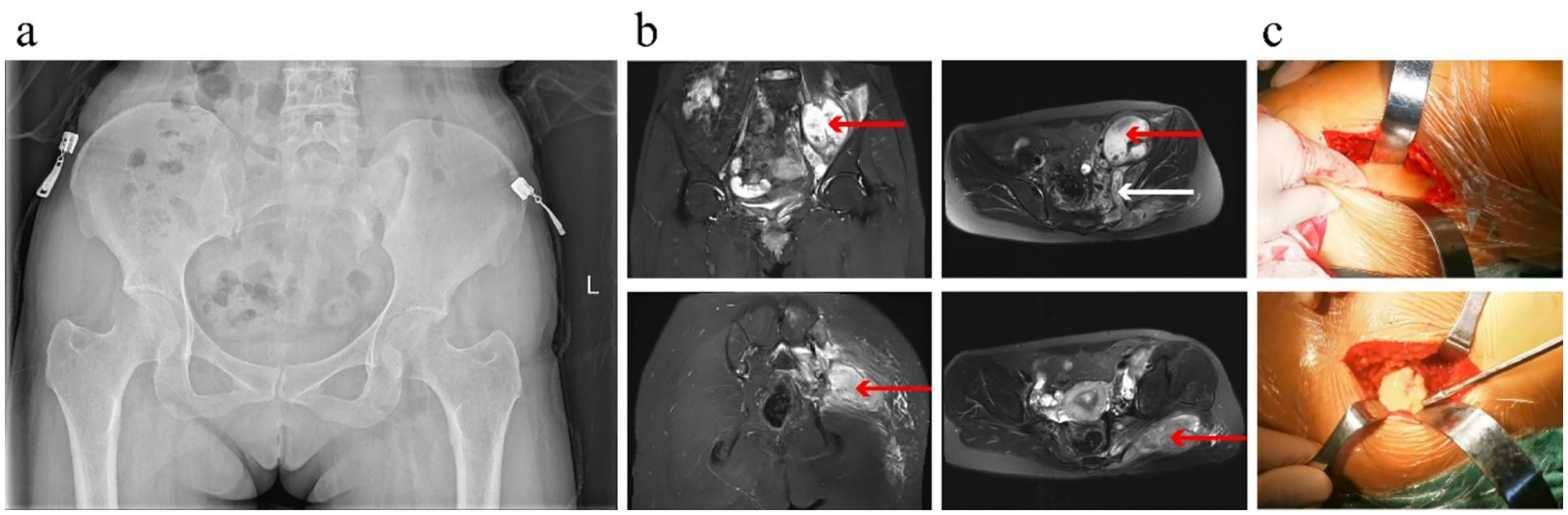
**(a)** X-ray examination shows no obvious abnormalities; **(b)** MRI reveals a mass measuring approximately 7.9 × 5.4 cm in the left iliac fossa and a 6.1 × 3.5 cm mass within the left gluteus maximus. The two lesions communicate through the left sacroiliac joint, with the iliac fossa abscess extending into the gluteal musculature. Abnormal signal changes are observed in the adjacent bone and soft tissues. Red arrows indicate the abscess lesions in the iliac fossa and gluteal region; black arrows indicate the communicating tract between the two regions; **(c)** Intraoperative findings show a large amount of yellow, turbid fluid mixed with caseous necrotic material within the iliac fossa and gluteal region.

A 31-year-old woman presented with left gluteal pain in May 2023, accompanied by mild radiating pain to the left lower limb and restricted range of motion in the left hip. She sought evaluation at an outside hospital, where symptomatic treatment failed to relieve her symptoms. Two months later, her gluteal pain progressively worsened, for which she self-administered analgesic medications. Later, the patient experienced intermittent and gradually progressive symptoms, primarily manifested as left gluteal discomfort and mild radiating pain in the lower limb. Because these symptoms were relatively mild and non-specific in the early stage, the patient initially sought care at an outside hospital, where she was misdiagnosed with lumbar disc herniation and received conservative treatment (Including anti-inflammatory and analgesic drugs, neurotrophic treatment, etc.), which failed to relieve her symptoms. In February 2025, she visited our hospital. Musculoskeletal ultrasound revealed a cystic–solid lesion within the left gluteus maximus muscle that communicated with the hip joint cavity. Magnetic resonance imaging (MRI) suggested infectious lesions involving the left iliac fossa and left gluteal region. She was admitted with a preliminary diagnosis of “left hip lesion.” The patient reported no history of hypertension, diabetes, or other chronic diseases and was not on any long-term medications. She denied any history of hepatitis, tuberculosis, malaria, or exposure to endemic areas, and reported no smoking or alcohol consumption. Notably, she had undergone UTND at an outside institution in March 2023, after which she experienced intermittent abdominal discomfort and one pregnancy loss. In addition, during the infertility evaluation, there were no clinical, laboratory, or imaging findings suggestive of genital tuberculosis. Therefore, GTB was considered unlikely in this patient. Physical examination at admission revealed marked tenderness over the left gluteal region and left lower abdomen, along with mildly restricted movement of the left hip joint. Laboratory tests showed: WBC count 3.51 × 10^9^/L, CRP 3.18 mg/dL, NEU 2.29 × 10^9^/L, ESR 53 mm/h, interleukin-6 12.3 pg/mL, and D-dimer 0.63 mg/L. The timeline of the patient’s treatment is shown in Figure 1. Admission radiographs and MRI findings are shown in Figures 2a,b.

### Surgical procedure

After admission, the patient underwent debridement of the left gluteal region and left iliac fossa. A groin incision was made over the left hip, and the iliacus muscle was bluntly dissected to separate the surrounding tissues until the cystic capsule was reached. Upon opening the capsule, approximately 100 mL of yellow, turbid fluid mixed with caseous necrotic material was released (Figure 2c). Specimens were collected for bacterial culture and histopathological examination. The cavity was extensively irrigated with normal saline, diluted povidone-iodine, and hydrogen peroxide, followed by curettage of the necrotic tissue. After a second round of copious irrigation with the same solutions, part of the cyst wall was excised. The cavity was soaked in concentrated povidone-iodine for 15 min and then packed with povidone–iodine–soaked gauze to prevent reflux of gluteal abscess contents. A second longitudinal incision was made on the lateral aspect of the left gluteal region. The gluteus maximus muscle was bluntly dissected to expose the cystic lesion. Upon opening the capsule, approximately 50 mL of thick, yellow, turbid fluid mixed with caseous debris was discharged (Figure 2c). Samples were collected for microbiological culture and pathological evaluation. The cavity was thoroughly irrigated with large volumes of normal saline, diluted povidone-iodine, and hydrogen peroxide, followed by meticulous curettage. After repeated irrigation with the same solutions, the cyst wall was completely excised. Exploration confirmed full clearance of the lesion and communication with the iliac fossa cavity. After changing gloves and drapes, hydrogen peroxide, diluted povidone–iodine, and normal saline were used for final irrigation of the wounds and deep tissues. Once clearance was confirmed, the deep and subcutaneous layers were closed with interrupted sutures, and the incision was closed in layers, followed by sterile dressing. Based on the gross intraoperative appearance, tuberculosis was strongly suspected. Empirical antimicrobial therapy with intravenous vancomycin and levofloxacin sodium was initiated postoperatively.

### Postoperative course

**Figure 3:**
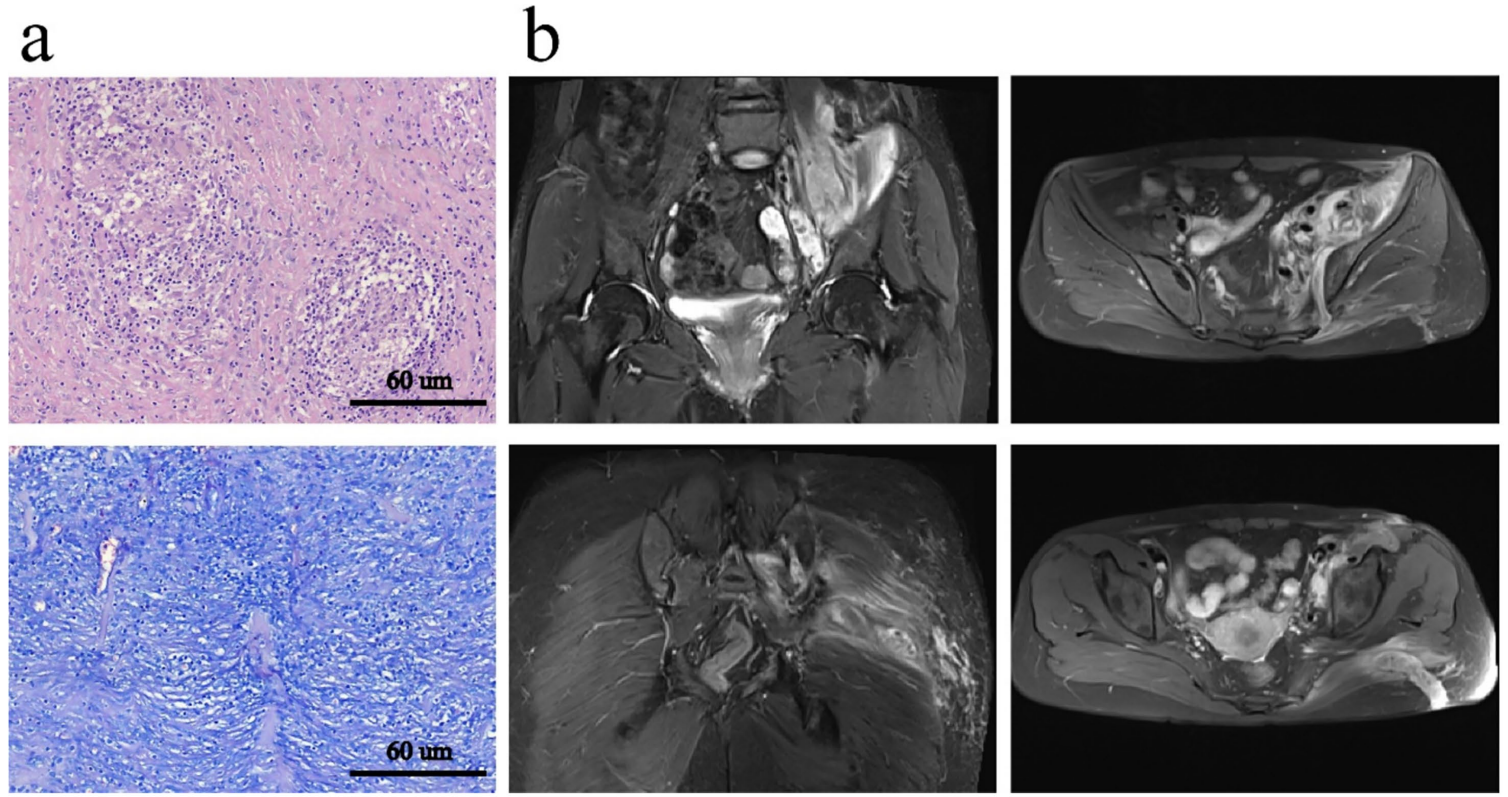
**(a)** Granulomatous inflammation of the left gluteal region and iliac fossa with extensive necrosis. Acid-fast staining shows suspicious positive bacilli, consistent with tuberculosis (×200); **(b)** MRI demonstrates swelling of the left iliopsoas and left gluteus maximus muscles with patchy abnormal signal intensity, with a reduced extent compared with previous imaging.

Postoperative laboratory evaluation showed a gradual decline in inflammatory markers. Blood culture demonstrated no bacterial growth after 72 h, and Brucella culture was negative. Repeat laboratory tests revealed: WBC 3.51 × 10^9^/L, NEU 2.29 × 10^9^/L, and CRP 3.18 mg/dL. Histopathological examination of the surgical specimen is shown in Figure 3a. The patient was started on anti-tuberculosis therapy consisting of rifampicin, isoniazid, ethambutol, and pyrazinamide. Postoperative MRI reexamination at 1 week is shown in the Figure 3b. She experienced no postoperative discomfort and was subsequently discharged.

## Discussion

This report describes a rare case of tuberculous abscess formation in the left iliac fossa and gluteus maximus following UTND. Tuberculosis is most commonly transmitted through airborne droplets, whereas osteoarticular and soft-tissue TB typically results from hematogenous dissemination (10–12). In the present case, the patient exhibited neither clinical symptoms nor imaging evidence of pulmonary tuberculosis, and she denied any history of TB exposure. She had undergone transvaginal oocyte retrieval at an outside facility in 2023, after which she developed discomfort in the left lower abdomen and left buttock, followed by a spontaneous abortion, yet no definitive diagnosis was reached for an extended period. Considering the clinical course and surgical history, the infection was suspected to be associated with the ART. Transvaginal oocyte retrieval is a puncture-based technique in which the needle traverses the vaginal fornix and paracervical tissues (8). In cases of unrecognized pelvic tuberculosis, the puncture pathway may provide a direct conduit for *M. tuberculosis* inoculation. Notably, no histopathological, microbiological, or radiological evidence of pelvic or fallopian tube tuberculosis was identified during the patient’s prior infertility workup, making overt genital tuberculosis less likely. However, given the well-documented insidious and subclinical nature of genital tuberculosis, the presence of a latent or minimally active pelvic lesion cannot be completely excluded. Therefore, two potential mechanisms should be considered. First, the transvaginal puncture procedure may have facilitated mechanical dissemination of a pre-existing, silent pelvic tuberculosis focus along the needle tract. Second, although rare, iatrogenic inoculation during the procedure remains a theoretical possibility. In addition, physiological stress associated with assisted reproductive techniques and subsequent pregnancy loss may have contributed to the reactivation of a latent tuberculosis infection (LTBI), further promoting disease progression. These possibilities highlight the complex interplay between procedural factors and host immune status in the pathogenesis of such rare presentations. In this patient, the pathogen was likely introduced along the needle tract and subsequently spread through surrounding tissues, leading to the development of an iliac fossa abscess that extended along anatomical planes. The purulent collection later tracked through the loose connective tissue adjacent to the greater sciatic foramen, eventually forming a secondary abscess within the gluteus maximus. This pattern of spread is consistent with the intermuscular extension routes described by Xu et al. (13) and Li et al. (14).

The diagnosis in this case was challenging due to the non-specific early symptoms of iliac fossa and gluteus maximus abscesses, which often result in delayed recognition (15, 16). Initially, the patient presented with left gluteal pain and mild radiating pain to the lower limb, symptoms that can easily be misattributed to lumbar disc herniation, piriformis syndrome, or non-specific arthritis. Radiographically, plain X-rays offered limited diagnostic value in the early stage, whereas MRI clearly delineated the abscesses within the left iliac fossa and gluteus maximus, demonstrating their communication through the left greater sciatic foramen into the gluteal musculature. Despite the strong indication of infection on imaging, definitive diagnosis relied on microbiological or histopathological confirmation. Postoperative histopathology of the abscess revealed granulomatous inflammation with necrosis and suspicious acid-fast bacilli, providing crucial evidence to guide anti-tuberculosis therapy. In this case, management involved a combination of anti-tuberculosis chemotherapy and surgical intervention. Once the diagnosis was confirmed, standardized anti-tuberculosis therapy was initiated, typically comprising a four-drug regimen of isoniazid, rifampicin, ethambutol, and pyrazinamide (17, 18). The recommended treatment duration is usually 9–12 months or longer to ensure complete bacterial eradication and prevent recurrence. Given the large size of the iliac fossa abscess in this patient (approximately 7.9 × 5.4 cm), achieving effective intralesional drug concentrations with medication alone was challenging. Moreover, the thick abscess wall and the presence of necrotic tissue further impair drug penetration. Therefore, the primary objective of surgical intervention is to reduce the local microbial burden, and it must be followed by appropriate pharmacological therapy to achieve effective infection control. Surgical debridement rapidly reduces bacterial load and relieves local pressure. For tuberculous abscesses that respond poorly to medical therapy or demonstrate recurrent episodes, early consideration of surgical intervention is warranted. Close postoperative follow-up is essential, with dynamic evaluation of therapeutic efficacy based on clinical symptoms, inflammatory markers (CRP and ESR), and imaging modalities (ultrasonography or MRI), allowing timely adjustment of the treatment strategy.

Definitive diagnosis of such infections requires an integrated approach, combining clinical evaluation, imaging studies (such as plain radiography and MRI), and microbiological or histopathological confirmation via surgical or needle biopsy. Anti-tuberculosis chemotherapy remains the cornerstone of treatment; however, for patients with large abscesses or those who do not respond to medical therapy, prompt and thorough surgical debridement with drainage is indicated. Given the high risk of recurrence, long-term postoperative monitoring and follow-up are essential. With the increasing use of ART in recent years (19, 20), which often involve invasive procedures, attention should be paid to the potential association between reproductive interventions and tuberculosis. It is recommended that patients undergo systematic tuberculosis screening prior to ART procedures to minimize the risk of severe complications such as procedure-related tuberculous abscesses.

## Conclusion

We report a rare case of tuberculous abscess involving the left gluteal region and iliac fossa following UTND. In regions where tuberculosis is endemic, clinicians should consider TB in the differential diagnosis for patients with a history of invasive procedures who present with unexplained musculoskeletal pain or deep-seated abscesses, even in the absence of typical constitutional symptoms or pulmonary involvement.

From the patient’s perspective, the onset of symptoms after the procedure was initially mild and non-specific, which made it difficult to recognize the underlying cause. The persistent gluteal pain and prolonged period without a definitive diagnosis caused considerable anxiety and uncertainty. After being admitted to our hospital and receiving a clear diagnosis followed by surgical treatment and standardized anti-tuberculosis therapy, the patient experienced significant relief of symptoms and gradual recovery. She expressed appreciation for the medical team’s efforts and emphasized the importance of timely diagnosis and appropriate treatment for similar conditions. The patient also hopes that sharing her experience may help increase awareness of rare procedure-related infections and assist other patients in receiving earlier diagnosis and management.

## Data Availability

The original contributions presented in the study are included in the article/supplementary material, further inquiries can be directed to the corresponding author.
